# Coordinated epigenomic reprogramming and chromatin-mediated transcriptional regulation during soybean callus development

**DOI:** 10.3389/fpls.2026.1905128

**Published:** 2026-09-25

**Authors:** Yeong-Il Jeong, Min-Gyun Jeong, Joo-Seok Park, Yoram Choi, Jin-Hyun Kim, Si-hoo Park, Hong-Kyu Choi

**Affiliations:** 1 Department of Applied Bioscience, Dong-A University, Busan, Republic of Korea; 2 SEEDERS lnc., Jung-gu, Daejeon, Republic of Korea; 3 Research Institute of Molecular Alchemy, Gyeongsang National University, Jinju, Republic of Korea; 4 Department of Agricultural Biotechnology, National Institute of Agricultural Sciences, RDA, Jeonju, Republic of Korea; 5 Department of Biomaterials Engineering, Dong-A University, Busan, Republic of Korea

**Keywords:** callus induction, chromatin accessibility, dedifferentiation, histone modification, open chromatin regions (OCRs), soybean, transcription factor networks

## Abstract

**Introduction:**

Callus formation is a fundamental developmental process involving cellular dedifferentiation and regeneration in plants. However, the epigenetic mechanisms underlying this process remain poorly understood.

**Methods:**

Here, we performed integrative analyses of time-series ATAC-seq, ChIP-seq (H3K27ac and H3K27me3), and transcriptome datasets to investigate chromatin-mediated transcriptional regulation during soybean callus development.

**Results:**

Genome-wide profiling identified extensive epigenetic remodeling during early callus induction, including 15,740 differentially accessible regions (DARs) associated with dynamic transcriptional changes. Distinct chromatin accessibility patterns corresponded to sequential developmental programs, including repression of explant identity, activation of hormone-responsive cell proliferation pathways, and stress-associated responses during later stages. Chromatin accessibility and H3K27ac were strongly associated with transcriptional activation during early induction, whereas *de novo* open chromatin regions (OCRs) were closely associated with increased expression of key developmental regulators. Motif enrichment and chromVAR analyses further identified stage-specific TF motif-associated regulatory signatures involving the ARF–LBD, ERF, E2F/DP, and BES1 families during early proliferative reprogramming, followed by WOX- and WRKY-associated signatures during callus maintenance and stress adaptation.

**Discussion:**

Together, our findings suggest that soybean callus formation is accompanied by hierarchical epigenetic reprogramming involving dynamic chromatin remodeling and stage-specific TF motif-associated regulatory signatures, providing a genome-wide framework for understanding plant cellular reprogramming and improving regeneration-based crop biotechnology.

## 1 Introduction

Plant tissue culture has advanced considerably since Haberlandt first proposed the concept of cellular totipotency in 1902 (Haberlandt, 1902). It is now widely applied in diverse areas, including genetic transformation, accelerated breeding, and genome editing-based crop improvement (Bidabadi and Mohan Jain, 2020). Among various plant tissue culture systems, callus represents a central experimental model for studying cellular plasticity. Callus formation is typically triggered by external stimuli such as wounding or pathogen infection and involves the proliferation of relatively undifferentiated cells with cellular totipotency (Ikeuchi et al., 2013). Importantly, callus tissue retains the capacity for organogenesis or somatic embryogenesis, making it a powerful system for investigating cell-fate transitions and transcriptional reprogramming during dedifferentiation and regeneration (Fehér, 2019; Ikeuchi et al., 2013; Su et al., 2021).

In our previous study, we characterized the transcriptional landscape underlying soybean callus formation using high-resolution time-series RNA-seq analysis (Park et al., 2023). Gene expression profiling across seven time points (0–42 days after induction, DAI) revealed that 50.31% of expressed genes undergo significant transcriptional changes during callus induction. Clustering analysis of 27,639 differentially expressed genes identified distinct temporal expression patterns associated with key biological processes, including early wounding responses, hormone biosynthesis and signaling, cell cycle activation, and cell wall remodeling. Notably, auxin, cytokinin, and brassinosteroid signaling pathways exhibited coordinated activation patterns linked to meristematic fate acquisition and cell proliferation. These findings revealed sequential changes in transcriptional programs associated with soybean callus development. However, RNA-seq has limitations in understanding the reorganization of the regulatory chromatin landscape associated with these transcriptional transitions. Therefore, resolving the chromatin-level changes associated with transcriptional reprogramming requires an integrated analysis of epigenomic and transcriptomic dynamics throughout callus development.

In eukaryotes, chromatin accessibility and histone modifications are fundamental regulators of gene expression, controlling transcriptional activation and repression through modulation of nucleosome occupancy and positioning, regulatory cis-element accessibility, and higher-order chromatin organization (Marand et al., 2023; Chen et al., 2024). Accessible chromatin regions provide binding platforms for transcription factors and co-regulators, enabling context-dependent gene regulation in response to developmental and environmental cues (Zhou et al., 2021; Candela-Ferre et al., 2024). In parallel, post-translational histone modifications contribute to transcriptional regulation; for example, histone H3 lysine 27 acetylation (H3K27ac) is associated with active enhancers and promoters, whereas trimethylation at the same residue (H3K27me3) is linked to transcriptional repression and chromatin compaction, often mediated by Polycomb group complexes (Creyghton et al., 2010; Du et al., 2013; Shen et al., 2021).

Recent studies in plants have demonstrated that callus formation and cellular dedifferentiation are accompanied by extensive epigenetic reprogramming. During callus induction, repressive histone marks such as H3K27me3 are reduced at developmental regulatory loci, while active marks including H3K4me3 are enriched at genes associated with meristem identity and cell cycle progression (Zhao et al., 2020; Zheng et al., 2022). In addition, phytohormone signaling pathways influence chromatin states by modulating chromatin accessibility and remodeling, thereby linking external cues such as wounding, auxin, and cytokinin to dynamic transcriptional regulation (Potter et al., 2018; Kuhn et al., 2020; Shao et al., 2024). Consistent with these findings, ATAC-seq and integrative epigenomic studies in *Arabidopsis* and wheat have revealed stage-specific changes in chromatin accessibility and epigenomic states during plant development and regeneration (Wang et al., 2020; Liu et al., 2023a; Zhao et al., 2023; Chen et al., 2025). However, despite these advances, how the relationships among chromatin accessibility, active and repressive histone modifications, and transcriptional programs change across successive stages of callus development remains incompletely understood. In particular, stage-dependent changes in these regulatory relationships from callus induction through rapid proliferation to the stationary stage have not been systematically characterized in soybean.

In this study, we investigated the chromatin architecture associated with soybean callus formation using ATAC-seq and ChIP-seq. ATAC-seq was employed to map genome-wide chromatin accessibility and identify open chromatin regions (OCRs), while ChIP-seq targeting H3K27ac and H3K27me3 was used to profile active and repressive histone modifications, respectively. We examined key developmental transitions from explant tissue (DAI 0) through early callus induction and proliferation (DAI 4–7) to the stationary stage (DAI 14–28). By integrating these epigenomic datasets with our previously generated RNA-seq data (Park et al., 2023), we examined the temporal relationships among chromatin accessibility, histone modifications, and transcriptional changes during callus development. Our integrative analysis revealed distinct stage-dependent associations between epigenomic states and transcription. During early callus induction, *de novo* chromatin opening was associated with transcriptional activation, while chromatin accessibility and H3K27ac showed positive correlations with gene expression. In contrast, the associations between histone modifications and transcription became substantially weaker during later developmental stages. These findings suggest that the relationships between epigenomic states and transcription change across developmental stages, providing chromatin-level insights into transcriptional reprogramming during soybean callus development.

## 2 Materials and methods

### 2.1 Plant materials and callus induction

Soybean (*Glycine max* L. Merr, cv. Kwang-an) seeds were used for callus induction. Seeds were sterilized using chlorine gas and subsequently imbibed in 12 ml of deionized water (DIW) per 150 seeds for 24 h. The seeds were then washed with 1% (v/v) sodium hypochlorite (bleach) solution, followed by three rinses with autoclaved DIW, and incubated in 50 ml conical tube filled with sterile water for an additional 24 h.

Following soaking, the seeds were bisected using a sterile scalpel (#11 blade), and the embryonic axes were removed. The cotyledon halves were wounded by making ten scratches across the surface in multiple directions with the scalpel. In addition, the hypocotyl–cotyledon junction was scratched in a direction perpendicular to the cotyledon (Jiang et al., 2010; Park et al., 2023). The wounded explants were then transferred onto callus induction medium (CIM; Gamborg’s B5 medium) supplemented with 3 mM MES, 3% (w/v) sucrose, 8 mg/L 2,4-D, 1 mg/L 6-benzyladenine (BA), and 250 mg/L cefotaxime. Explants were cultured at 28 °C under continuous darkness and subcultured onto fresh CIM every two weeks.

Callus tissues containing the meristematic region together with small portions of the cotyledon and hypocotyl were collected at 4, 7, 14, and 28 days after induction (DAI). Non-wounded explants at DAI 0 were used as controls. For each time point, three independent biological replicates were prepared from pooled explants for ATAC-seq and ChIP-seq library construction.

### 2.2 ATAC-seq library preparation and sequencing

For ATAC sequencing, 1 g of callus tissue from each time point (DAI 0, 4, 7, 14 and 28) was incubated in 37 ml of cross-linking buffer (0.4 M sucrose, 10 mM Tris-HCl, 10 mM Na-Bu, 0.1 mM PMSF, 5 mM 2-mercaptoethanol, 1% formaldehyde) under vacuum infiltration at room temperature for 10 minutes. Cross-linking was quenched by adding 2.5 mL of 2 M glycine solution, followed by three washes with sterilized distilled water (DW).

Samples were flash-frozen in liquid nitrogen and ground into a fine powder. Nuclei were isolated using the CelLytic PN Plant Nuclei Isolation/Extraction Kit (Sigma-Aldrich, St. Louis, MO, USA), and stained with DAPI (4′,6-Diamidino-2-phenylindole dihydrochloride). Nuclei were quantified using a hemocytometer, and approximately 50,000 nuclei were treated with Tn5 transposase (Illumina, San Diego, CA, USA) at 37 °C for 30 min.

Reverse cross-linking was performed using NaCl (final concentration 0.2 M) at 65 °C overnight (~ 16h), followed by RNase A and Proteinase K treatment and DNA was purified using the MinElute PCR Purification Kit (Qiagen, Hilden, Germany). Paired-end libraries were sequenced on the Illumina NovaSeq 6000 platform (Macrogen, Seoul, Republic of Korea) with three biological replicates per condition.

### 2.3 ChIP-seq library preparation and sequencing

For ChIP-seq, 1 g of callus tissue per sample was cross-linked, and nuclei were isolated as described above. Chromatin was sheared to 250–500 bp fragments using a Bioruptor sonicator (30 s ON/30 s OFF, High setting, 15 min). Immunoprecipitation was performed with 2.5 μg of antibodies against H3K27ac (Abcam, Cambridge, UK; ab4729) and H3K27me3 (Abcam; ab6002), followed by overnight incubation (~16 h) at 4 °C with continuous rotation.

The antibody–chromatin complexes were captured with a mixture of Protein A and Protein G magnetic beads (Bio-Rad, Hercules, CA, USA), followed by overnight incubation (~16 h) at 4 °C with continuous rotation. The bead-bound chromatin complexes were sequentially washed with low-salt wash buffer (0.1% SDS, 0.1% Triton X-100, 2 mM EDTA, 20 mM Tris-HCl [pH 8.0], and 150 mM NaCl), high-salt wash buffer (0.1% SDS, 0.1% Triton X-100, 2 mM EDTA, 20 mM Tris-HCl [pH 8.0], and 500 mM NaCl), LiCl wash buffer (250 mM LiCl, 1% NP-40, 1% sodium deoxycholate, 1 mM EDTA, and 10 mM Tris-HCl [pH 8.0]), and TE buffer (10 mM Tris-HCl [pH 8.0] and 1 mM EDTA). Chromatin was eluted twice from the magnetic beads using elution buffer (1% SDS and 0.1 M NaHCO_3_). Cross-links were reversed using NaCl (final concentration, 0.2 M) at 65 °C overnight (~16 h), followed by RNase A and Proteinase K treatment. DNA was purified using phenol–chloroform–isoamyl alcohol (PCI) extraction. Sequencing libraries were prepared and subjected to paired-end sequencing on the Illumina NovaSeq 6000 platform (Macrogen, Seoul, Republic of Korea), with three biological replicates for each time point.

### 2.4 Analysis of RNA-seq, ATAC-seq and ChIP-seq data

All sequencing reads were aligned to the soybean reference genome (Wm82.a4.v1). For ATAC-seq, ChIP-seq data, reads were aligned using BWA-MEM (Li and Durbin, 2009) program with default parameters. For quality assessment, peak calling was performed on individual replicates, and the resulting peak numbers, together with FRiP scores and mapping statistics, were used to assess library quality (**Supplementary Table 1**). Libraries were retained unless they were discordant with the other replicates of the same time point, showed an aberrant FRiP for the mark assayed, or yielded an anomalous number of peaks relative to their replicates (**Supplementary Table 1**). Two retained replicates per histone mark and time point were then merged to improve peak-calling sensitivity.

The merged bam files were converted into bigwig files using bamCoverage and bigwigCompare, and then visualized using computeMatrix, plotProfile and plotHeatmap from deepTools (Ramírez et al., 2016). Peak calling was performed using MACS3 (Zhang et al., 2008) with parameters “callpeak--format BAMPE --bdg --nolambda --keep-dup all –g 769565120.0”, with the additional parameter’-broad’ for methylation. Peaks were annotated using the ChIPSeeker R package (Yu et al., 2015).

ATAC-seq peaks, defined as open chromatin regions (OCRs) were quantified using HTSeq (Anders et al., 2015) and normalized using DESeq2 R package (Love et al., 2014). Differentially accessible regions (DARs) were identified relative to DAI 0 using a threshold of log_2_ fold change ≥ 1. Temporal clustering of DARs was performed using the Mfuzz R package (Kumar and Futschik, 2007), with all DARs retained and no membership cutoff applied.

RNA-seq data from our previous study (Park et al., 2023) were reanalyzed using HISAT2 for alignment (Kim et al., 2019), and HTSeq and DESeq2 for quantification. Differentially expressed genes (DEGs) were defined using |log_2_ fold change| ≥ 2 and false discovery rate (FDR) < 0.05. Principal component analysis (PCA) was conducted using regularized log-transformed (rlog) counts. Heatmaps of DEGs and DARs were generated using pHeatmap R package (Kolde, 2015). Associations among chromatin accessibility, histone modification and transcription were summarized as Pearson correlation coefficients between cluster-level mean Z-score profiles, computed across the five time points of the full time course and across the three time points within each phase. Spearman rank correlations were computed in parallel and are reported alongside the Pearson values in **Supplementary Table 4**.

### 2.5 Gene ontology and transcriptional regulation analysis

Transcription factor (TF) binding motifs were obtained from PlantTFDB v4.0 (Jin et al., 2017) in MEME format. Gene ontology (GO) enrichment analyses for OCR-associated and TF-target genes were conducted using clusterProfiler R package (Wu et al., 2021) and PlantRegMap.

Position weight matrices (PWMs) for soybean TFs were obtained from PlantTFDB v4.0. TF binding sites within OCRs were identified using FIMO v5.5.5 (Grant et al., 2011) from the MEME Suite, with a significance threshold of *p* < 1 × 10^−5^. Only high-confidence motif matches were retained for downstream analysis. Motif enrichment analysis was performed using MEME-AME (McLeay and Bailey, 2010), and TF activity was inferred using the chromVAR R package (Schep et al., 2017).

### 2.6 Construction of gene regulatory network (GRN)

Gene expression levels were normalized using DESeq2 and transformed into regularized log (rlog) values. Genes and TFs with rlog ≥ 3 were considered actively expressed, with an optional TPM (Transcripts Per Million) ≥ 1 threshold applied for stringent filtering.

TF binding sites within OCRs were assigned to putative target genes when the site overlapped the gene’s promoter region, defined as upstream 2,000 bp from transcription start site (TSS).

Regulatory interactions (TF - target edges) were defined by integrating multiple data layers: (i) Motif-Peak Association: TF motif presence within OCRs (FIMO *p* < 1 × 10^−5^), (ii) Peak-Gene Linkage: assignment of each TF binding site to a target gene when the site overlapped the gene’s promoter region, and (iii) Expression Co-occurrence: active expression of both the TF and its target gene (rlog ≥ 3, and/or TPM ≥ 1). Edges were not restricted to TF–target pairs from the same expression cluster Target genes were organized according to the gene expression clusters defined in our previous study (Park et al., 2023) to build module-level regulatory subnetworks, allowing a given TF to regulate target genes across different clusters.

Final TF-target pairs satisfying all criteria were used to construct the integrated regulatory network, with target genes organized by expression cluster. GO enrichment analysis was performed, and callus-related terms were retained. Networks were exported in GraphML format and visualized using Gephi (Bastian et al., 2009) with the ForceAtlas 2 layout algorithm to identify hub TFs and regulatory modules. Visualization parameters were manually optimized.

## 3 Result

### 3.1 Overview of epigenomic dynamics during soybean callus development

To investigate epigenomic regulation during soybean callus development, we first defined representative developmental stages based on our previous transcriptomic analysis (Park et al., 2023). In that study, time-series RNA-seq analysis identified extensive transcriptional reprogramming during callus formation and classified callus development into three distinct stages according to growth dynamics and morphology: pre-callus (DAI 0), callus-log (DAI 4–7), and callus-stationary (DAI 14–28). These stages were accompanied by characteristic morphological changes during callus growth (**Figure 1A**). Notably, the transition from explant tissue (DAI 0) to early callus formation (DAI 4–7) represents a critical cell fate reprogramming stage. Based on these developmental stages, representative time points (DAI 0, 4, 7, 14, and 28) were selected for epigenomic profiling.

**Figure 1:**
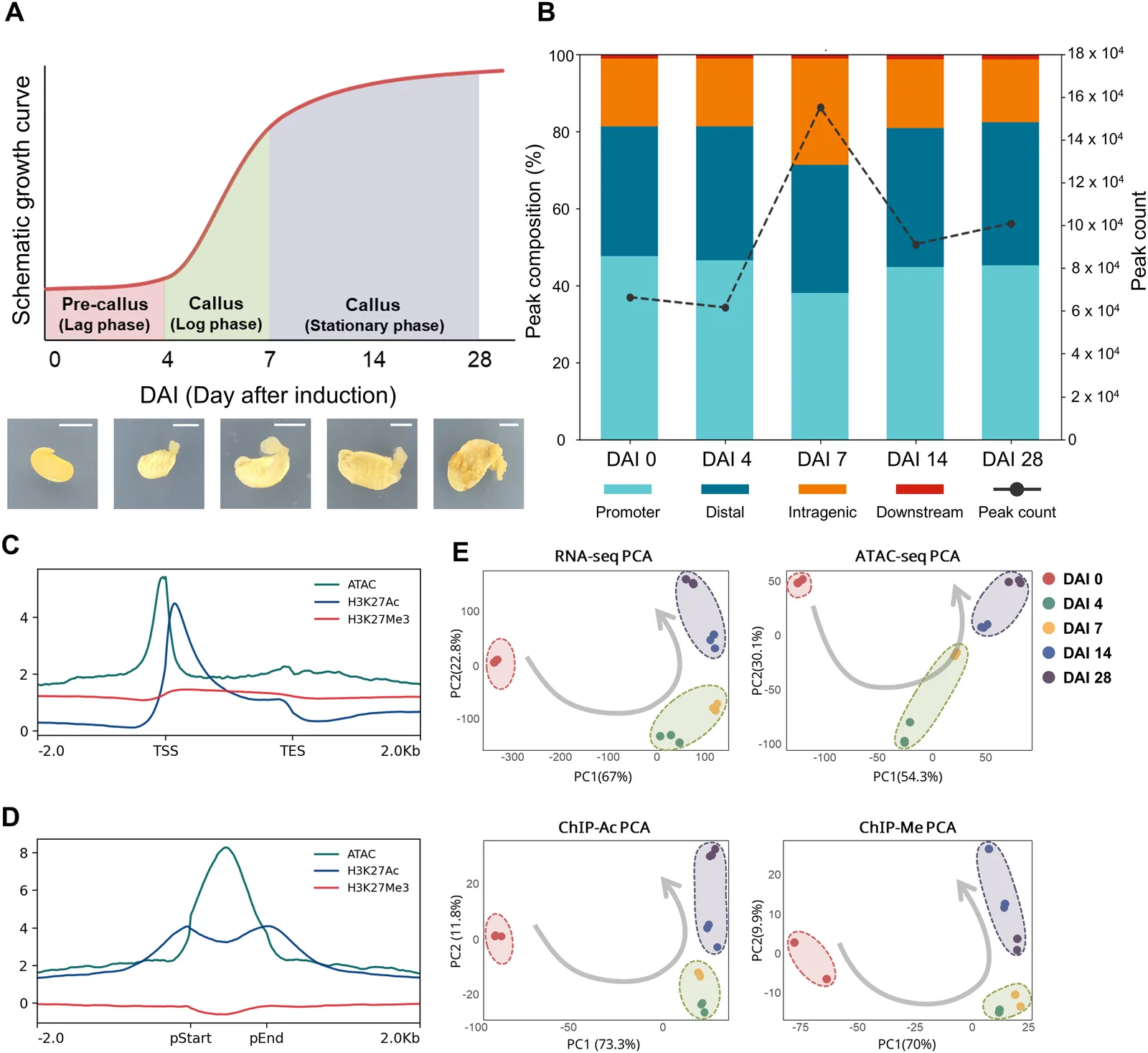
Epigenomic dynamics during soybean callus development. **(A)** Time-course progression of soybean callus development. The upper schematic illustrates the representative callus growth pattern and developmental stages based on our previous study (Park et al., 2023). The lower images show representative morphological changes during callus induction and development. White scale bars indicate 1 cm. **(B)** Genome-wide distribution of open chromatin regions (OCRs) across developmental stages. Stacked bars represent OCR composition (%), and the dashed line indicates the number of peaks. Signal profiles of epigenetic marks across genomic features [**(C)**; DAI 28, 28 days after induction] and OCR peaks [**(D)**; DAI 7]. **(E)** Principal component analysis (PCA) of transcriptomic and epigenomic datasets. Samples are grouped into three developmental stages: pre-callus, callus-log, callus-stationary, as defined in **(A)**. TSS, transcription start site; TES, transcription end site; pStart, peak start; pEnd, peak end.

To characterize stage-specific transcriptomic and epigenomic features, RNA-seq datasets corresponding to developmental stage were extracted from our previous study, and ATAC-seq and ChIP-seq analyses were performed to profile chromatin accessibility and histone modifications (H3K27ac and H3K27me3), respectively. In total, 15 ATAC-seq libraries and 49 ChIP-seq libraries were generated, comprising 30 immunoprecipitation and 19 input libraries. Ten immunoprecipitation libraries were excluded during quality filtering or to equalize replicate number across time points, leaving two replicates per histone mark and time point for the merged analyses (**Supplementary Table 1**). RNA-seq datasets were reanalyzed using the updated soybean reference genome (Wm82.a4.v1) following the same analysis workflow as in the previous study.

Peak calling of ATAC-seq and ChIP-seq data using MACS3 identified 61K–155K open chromatin regions (OCRs) (**Figure 1B**; **Supplementary Table 2**), 69K–78K H3K27ac peaks, and 7K–24K H3K27me3 peaks (**Supplementary Figure 1**). OCRs were predominantly enriched in promoter regions (45%), followed by distal intergenic regions (35%), intragenic regions (17%), and downstream regions (~1%). Notably, the total number of OCRs increased markedly at DAI 7 and was accompanied by an increase in intragenic OCRs (**Figure 1B**), indicating extensive chromatin remodeling during active callus proliferation. In contrast, H3K27ac peaks showed relatively stable genomic distributions across developmental stages, whereas H3K27me3 peaks progressively decreased until DAI 7, particularly within promoter regions, before recovering at DAI 14 (**Supplementary Figure 1**).

Reproducibility was assessed on individual biological replicates. Replicates from the same time point were consistently more similar to one another than to replicates from other time points (H3K27ac, Spearman rho = 0.93-0.97 within time points versus 0.89 overall), and clustered by developmental stage in principal component analysis (**Supplementary Figure 2**). Signal profiling demonstrated strong enrichment of OCR and H3K27ac signals around transcription start sites (TSSs), whereas H3K27me3 signals were broadly distributed across gene bodies (**Figure 1C**; **Supplementary Figure 3**). Moreover, H3K27ac signals displayed a characteristic bimodal enrichment pattern flanking OCR boundaries, while H3K27me3 signals were depleted within OCR regions (**Figure 1D**). To integrate chromatin accessibility with transcriptomic profiles, merged peak sets were generated using a weighted mean-based peak merging strategy and subjected to principal component analysis (PCA). PCA showed clustering of biological replicates according to developmental stage across the multi-omics datasets (**Figure 1E**). Across all datasets, the most pronounced separation was observed between DAI0 and DAI4, while the ATAC-seq dataset additionally exhibited a distinct separation between DAI4 and DAI7 (**Figure 1E**). These patterns suggest that the transition from explant to callus is associated with extensive epigenomic and transcriptional reprogramming.

### 3.2 Chromatin accessibility clusters define transcriptional programs during callus development

To characterize chromatin accessibility dynamics during callus development, differentially accessible regions (DARs) were identified from OCRs located in promoter (+500 to −2000 bp from the TSS) and distal regulatory regions (up to −10 kb from the TSS). A total of 15,740 DARs associated with 12,201 genes were identified and classified into six temporal accessibility clusters using the Mfuzz algorithm, and the resulting profiles were unchanged when restricted to core members (membership ≥ 0.7) (**Figure 2A**; **Supplementary Figure 4**; **Supplementary Figure 5**).

**Figure 2:**
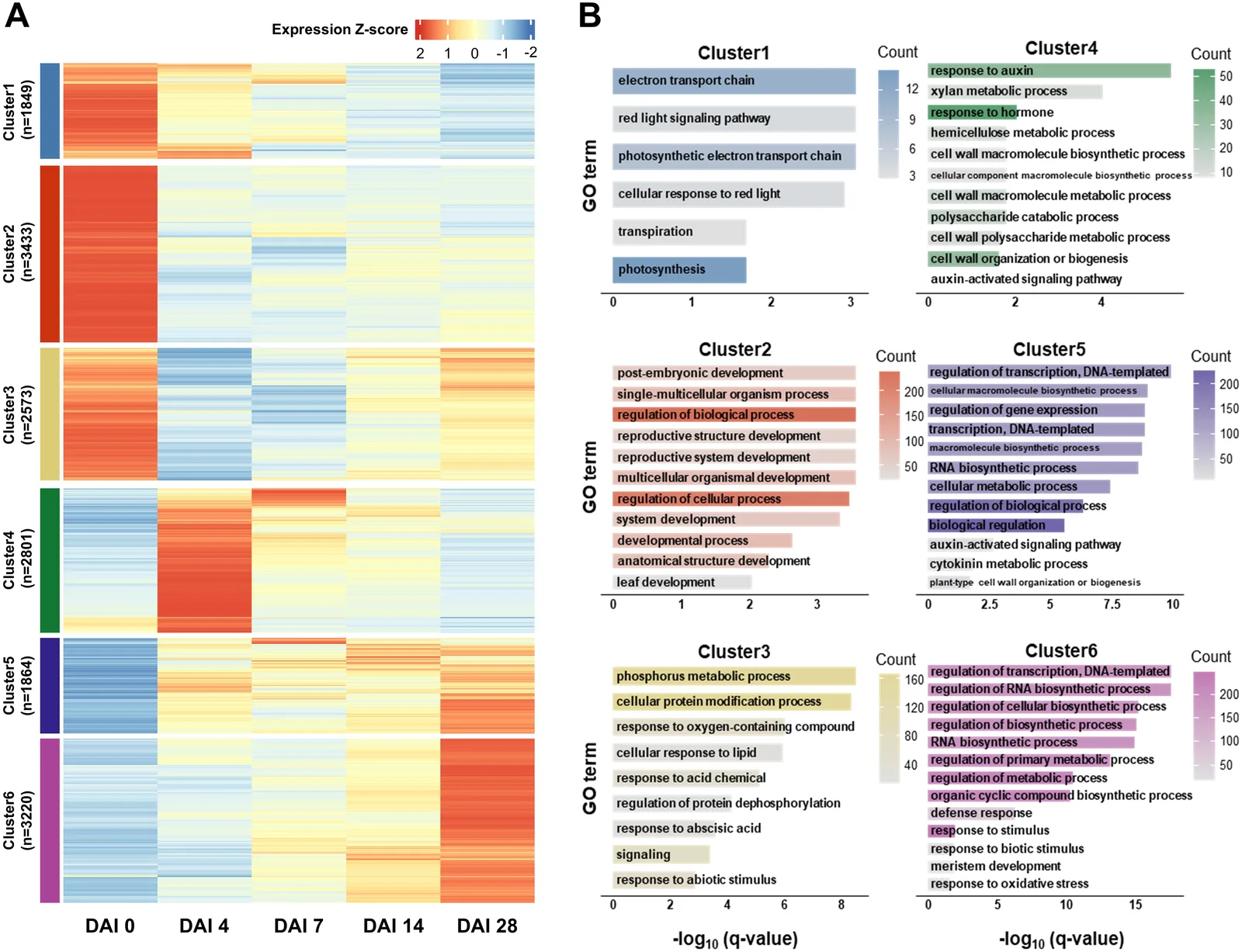
Dynamic chromatin accessibility clusters associated with transcriptional changes during callus development. **(A)** Heatmap exhibiting temporal accessibility patterns of 15,740 differentially accessible regions (DARs) identified during callus formation. DARs were grouped into six clusters using soft clustering based on standardized accessibility profiles. The color scale represents Z-score-normalized signal intensity. **(B)** Gene Ontology (GO) enrichment analysis of genes associated with each DAR cluster (Pearson correlation coefficient, *r* ≥ 0.3). Bar plots display representative enriched biological processes for each cluster.

To examine the functional significance of these accessibility patterns, GO enrichment analysis was performed using peak–gene associations exhibiting positive correlations between DAR accessibility and gene expression (Pearson correlation coefficient, *r* ≥ 0.3) (**Supplementary Table 3**). The resulting GO terms therefore represent biological processes associated with coordinated chromatin accessibility and transcriptional regulation (**Figure 2B**).

Clusters 1 and 2 exhibited high chromatin accessibility at the explant stage (DAI 0), followed by gradual or rapid decreases during callus induction. GO terms associated with these clusters were enriched for photosynthesis, reproductive development, and tissue maturation processes, suggesting progressive repression of explant- or organ identity programs during early dedifferentiation.

In contrast, Cluster 4 showed a marked increase in chromatin accessibility during the callus-log stage (DAI 4–7), coinciding with active cell proliferation. This cluster was enriched for auxin- and cytokinin-responsive genes (e.g., ARFs, Aux/IAAs, *PIN1*, and ARRs) as well as LBD family genes (*LBD15*, *LBD29*, and *LBD36*) implicated in callus induction. Genes associated with cell wall remodeling and cell cycle progression, including CYCs, EXPs, *PME1*, and XTHs, were also highly represented. These findings indicate that Cluster 4 corresponds to the early proliferative stage of callus formation associated with hormone signaling and cell wall reorganization. Similarly, Cluster 5 was enriched for hormone-related genes (e.g., *IAA4*, ARRs, and *LOG1*) together with LBD (*LBD4*, *LBD11*, and *LBD18*) and WOX (*WOX4* and *WOX5*) family genes. Unlike Cluster 4, chromatin accessibility in Cluster 5 continuously increased throughout development and remained elevated during the stationary stage, suggesting an association with the maintenance of callus identity and sustained proliferative capacity.

In contrast, Clusters 3 and 6 exhibited increased accessibility during the stationary stage (DAI 14–28) and were enriched for genes involved in ABA signaling, ROS regulation, and stress- and defense-related responses. Unlike Cluster 5, which displayed a sustained increase in chromatin accessibility beginning at DAI 4, Cluster 6 exhibited only modest changes during the callus-log stage before showing pronounced chromatin opening during the stationary stage. Together, these findings are associated with late-stage stress adaptation- and defense-related transcriptional programs.

### 3.3 Dynamic coordination between chromatin accessibility and gene expression during callus development

Because transcriptional regulation is governed by multiple chromatin features, including chromatin accessibility and histone modifications, we next investigated how these epigenomic layers are coordinated during callus development by integrating RNA-seq, ATAC-seq, and ChIP-seq datasets for H3K27ac and H3K27me3 (**Figures 3A, C**).

**Figure 3:**
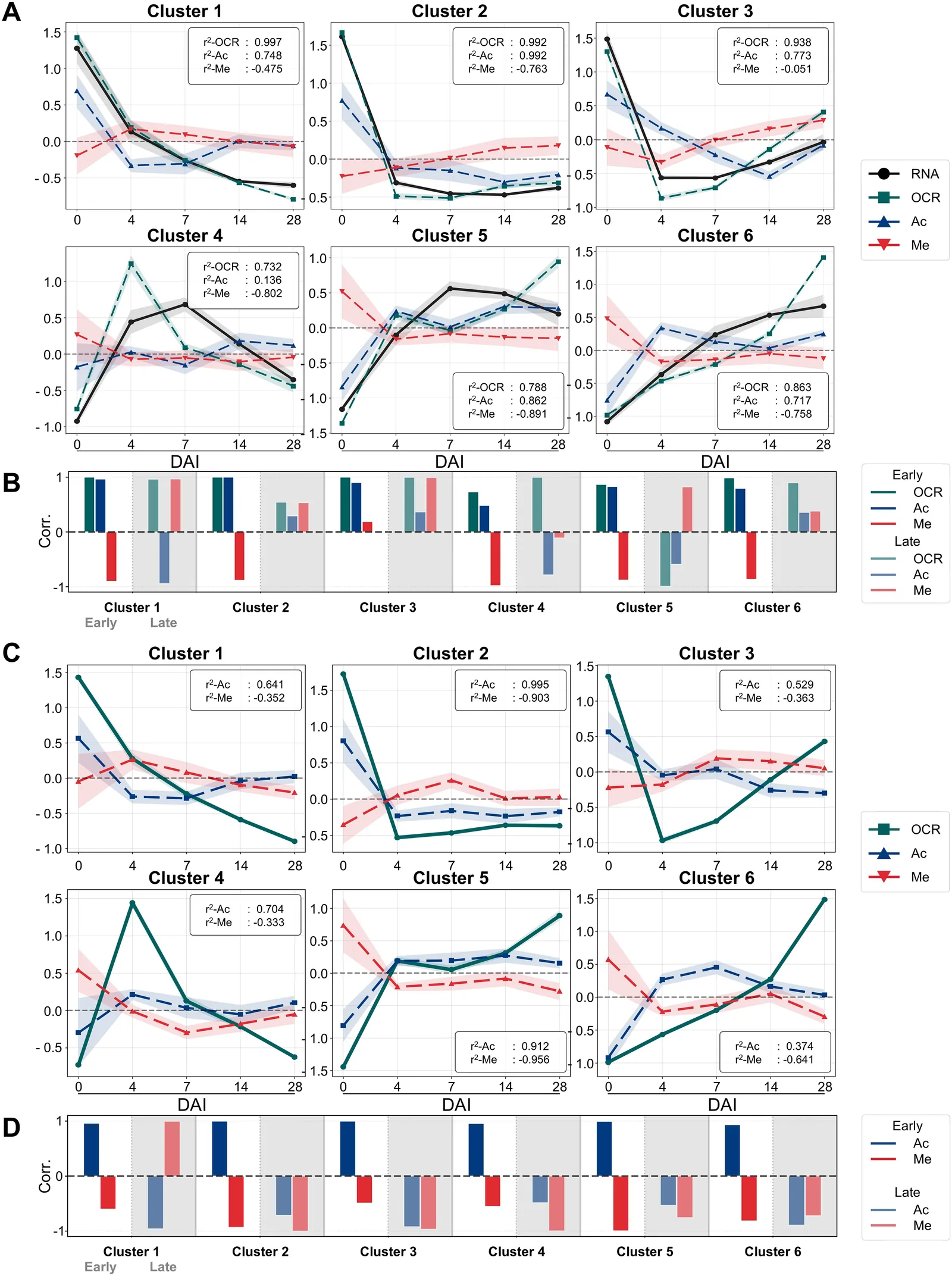
Coordinated chromatin states and gene expression during callus development. **(A)** Temporal dynamics of gene expression and epigenetic features across six clusters during callus development. Line plots represent standardized profiles of gene expression (black solid line), chromatin accessibility [open chromatin regions (OCRs); green dashed line], H3K27ac (blue dashed line), and H3K27me3 (red dashed line) across five developmental stages [days after induction (DAI) 0, 4, 7, 14, and 28]. **(B)** Pearson correlation analysis between gene expression and epigenetic signals. Bar plots indicate correlation coefficients between transcriptional levels and OCRs (green), H3K27ac (blue), and H3K27me3 (red). To highlight stage-dependent regulatory transitions, analyses were divided into Early (DAI 0-7; white background) and Late (DAI 7-28; dark gray background) phases. **(C)** Comparative temporal profiles of chromatin accessibility (OCR; green solid line) and histone modification signals. **(D)** Correlation analysis between chromatin accessibility and histone modifications.

H3K27ac and H3K27me3 represent histone marks associated with transcriptional activation and repression, respectively, and H3K27ac has been widely linked to enhancer and promoter activity (Du et al., 2013; Zheng et al., 2022). Correlation analysis revealed strong positive association between OCR accessibility and gene expression across most clusters (*r* ≥ 0.7), indicating that chromatin accessibility is closely linked to transcriptional activation during callus development (**Supplementary Table 4**). H3K27ac similarly showed positive correlations with gene expression in most clusters, consistent with its role as an active chromatin mark. In contrast, H3K27me3 exhibited predominantly negative correlations with gene expression (*r* ≤ −0.4), supporting its established function in transcriptional repression. Notably, Cluster 3 exhibited a relatively weaker negative correlation between H3K27me3 and gene expression, whereas Cluster 4 showed a relatively weaker positive correlation between H3K27ac and gene expression, particularly around DAI 7. These patterns suggest a transient regulatory transition during the shift from early induction to proliferative growth.

To further examine temporal regulatory dynamics, datasets were divided into Early (DAI 0–7) and Late (DAI 7–28) developmental phases (**Figures 3B, D**). During the Early phase, H3K27ac showed strong positive correlations with gene expression in Clusters 1, 4, and 5, whereas these associations weakened or became negative during the Late phase. Conversely, H3K27me3 exhibited positive correlations with gene expression in several clusters during the Late phase, suggesting a reduced or altered contribution of these epigenetic marks as transcriptional programs become stabilized.

Importantly, H3K27ac also showed strong positive correlations with chromatin accessibility across all clusters in the Early phase, indicating coordinated changes in H3K27ac enrichment and chromatin accessibility at enhancer- and promoter-associated regions during early callus induction. However, these associations weakened during later developmental stages, suggesting progressive stabilization of chromatin accessibility during later developmental stages. Equivalent rank-based (Spearman) coefficients gave the same direction of association in 88 of 90 comparisons, indicating that the relationships described here do not depend on the assumption of linearity (**Supplementary Table 4**). Together, these findings indicate stage-dependent shifts in the relationships among chromatin accessibility, H3K27ac, H3K27me3, and transcription during soybean callus development.

### 3.4 Chromatin accessibility remodeling during early callus induction is associated with transcriptional activation of developmental regulators

Given the stage-dependent epigenomic remodeling observed during callus development, we next investigated the relationship between changes in chromatin accessibility and transcriptional activation during early callus induction. To address this, ATAC-seq profiles at DAI 0 and DAI 4 were compared. A total of 3,454 OCRs identified across the two stages were classified into three categories based on their temporal accessibility patterns: (i) activated OCRs that newly emerged at DAI 4, (ii) deactivated OCRs that were present at DAI 0 but lost at DAI 4, and (iii) consistent OCRs that remained accessible at both stages (**Figure 4A**; **Supplementary Table 5**).

**Figure 4:**
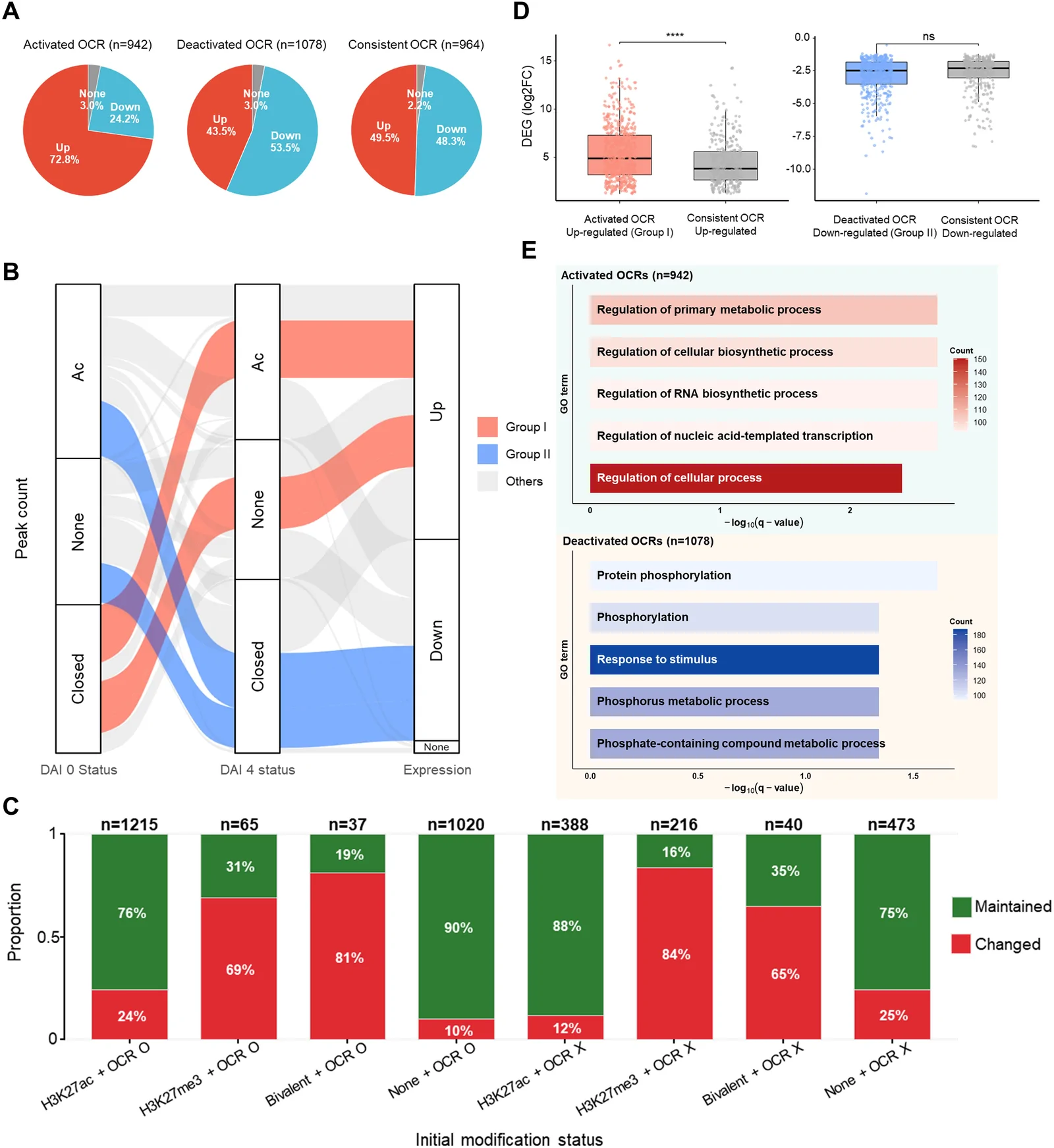
Association of early chromatin accessibility remodeling with transcriptional activation. **(A)** Pie charts showing the proportion of up-regulated (red), down-regulated (blue), and non-differentially expressed genes (gray) associated with Activated, Deactivated, and Consistent open chromatin regions (OCRs). **(B)** Sankey plot illustrating transitions in chromatin accessibility and histone modification states from 0 to 4 days after induction (DAI 0–4). Closed indicates regions lacking OCRs, None indicates OCRs without associated H3K27ac, and Ac indicates OCRs associated with H3K27ac. Flows represent the associations between chromatin states and gene expression changes (Up, Down). Group I (red flow) represents genes associated with newly activated OCRs (Activated OCRs) and transcriptional up-regulation, whereas Group II (blue flow) represents genes associated with loss of chromatin accessibility (Deactivated OCRs) and transcriptional down-regulation. **(C)** Bar plots displaying the stability of histone modification states. Proportion of maintained (green) and changed (red) histone marks is shown for regions with chromatin accessibility (OCR O) or without chromatin accessibility (OCR X). H3K27me3 and bivalent chromatin states exhibit higher plasticity than H3K27ac-marked regions. **(D)** Box plots comparing the magnitude of gene expression changes (log2 fold change) between Group I (Activated OCRs/Up-regulated genes) and Consistent OCRs/Up-regulated genes (left), and between Group II (Deactivated OCRs/Down-regulated genes) and Consistent OCRs/Down-regulated genes (right). Statistical significance was determined using Student’s t-test (****, p < 0.0001; ns, not significant). **(E)** GO enrichment analysis of genes in Group I (Activated OCRs) and Group II (Deactivated OCRs). Bar length represents enrichment significance [-log_10_(*q*-value)], and the color gradient indicates gene counts.

Integration of OCR dynamics with transcriptomic data revealed a strong association between chromatin accessibility gain and transcriptional activation. Among genes associated with activated OCRs, 72.8% of the 942 genes were up-regulated, whereas only 24.2% were down-regulated. In contrast, genes associated with deactivated OCRs (1,078 genes) showed predominantly reduced expression, with53.5% down-regulated relative to up-regulated genes. Genes associated with consistent OCRs (964 genes) exhibited relatively balanced expression patterns (**Figure 4B**). Consistent with these trends, Group I genes (activated OCRs with increased expression) showed significantly greater transcriptional induction than genes with consistent OCRs and increased expression. In contrast, Group II genes (deactivated OCRs with decreased expression) showed no significant difference in DEG magnitude compared with genes with consistent OCRs and decreased expression (**Figure 4D**). These findings indicate that *de novo* chromatin opening is strongly associated with transcriptional activation during early callus induction, whereas chromatin closure alone does not necessarily lead to immediate transcriptional repression.

To further characterize chromatin-state transitions associated with OCR remodeling, histone modification patterns surrounding OCRs were analyzed based on H3K27ac, H3K27me3, bivalent (regions marked by both H3K27ac and H3K27me3), or unmarked states (**Figure 4C**; **Supplementary Figure 6**). H3K27ac-associated OCRs and regions lacking both histone marks generally maintained stable chromatin states during callus induction. In contrast, H3K27me3-associated and bivalent regions exhibited substantially higher frequencies of chromatin-state transitions, including depletion of H3K27me3 signals. Although these regions represented a relatively small fraction of total OCRs, their dynamic behavior suggests that repressive and bivalent chromatin states possess elevated epigenetic plasticity during the early stage of cellular reprogramming.

To examine the biological significance of chromatin accessibility remodeling, GO enrichment analysis was performed for Group I (activated OCR) and Group II (deactivated OCR) (**Figure 4E**). Group I was enriched for biological processes associated with callus formation, including transcriptional regulation, developmental processes, and cellular metabolism. This group included genes involved in dedifferentiation and hormone signaling, such as *WOX4* (Glyma.06G041800), *ARF8* (Glyma.13G221400), and *IAA14* (Glyma.17G115300), all of which exhibited coordinated increases in chromatin accessibility and transcriptional activity. In contrast, Group II showed relatively weak enrichment for genes associated with stress responses and signal transduction, including stress-responsive transcription factors such as *NAC029* (Glyma.02G109800) and *WRKY15* (Glyma.01G189100), which were accessible and actively expressed at the explant stage but became repressed following callus induction.

Collectively, these results demonstrate extensive chromatin accessibility remodeling during early callus induction and show that the formation of *de novo* OCRs is closely associated with increased expression of developmental and hormone-responsive regulators implicated in cellular dedifferentiation and callus formation.

### 3.5 Temporal dynamics of transcription factor binding during callus induction

The coordinated changes observed between chromatin accessibility and gene expression during the transition from explant to callus suggested an association between TF motif-associated activity and stage-specific chromatin accessibility dynamics (**Figure 4**). To examine these regulatory dynamics, cluster-based TF motif enrichment analysis was performed using MEME-AME, and Temporal TF motif-associated activity was inferred using chromVAR (**Supplementary Table 6**).

Although TF binding motifs were broadly distributed across Clusters 2, 3, 5, and 6, TF families closely associated with callus induction and early cellular reprogramming, including ARF, LBD, ERF, E2F/DP, and BES1, were specifically enriched in Cluster 4 (**Figure 5A**). ARFs (Auxin Response Factors), central regulators of auxin signaling (Li et al., 2016), were highly enriched during the callus-log stage, suggesting an association between increased OCR accessibility at DAI 4 and auxin-responsive transcriptional programs. Consistent with this interpretation, motifs of the LBD (Lateral Organ Boundaries Domain) family, downstream effectors of ARFs involved in dedifferentiation and organ boundary formation (Xu et al., 2018), were also significantly enriched. The presence of callus-associated regulators such as *LBD16* and *LBD18* further supports an association with auxin-responsive developmental reprogramming during early callus formation (**Supplementary Figure 7**).

**Figure 5:**
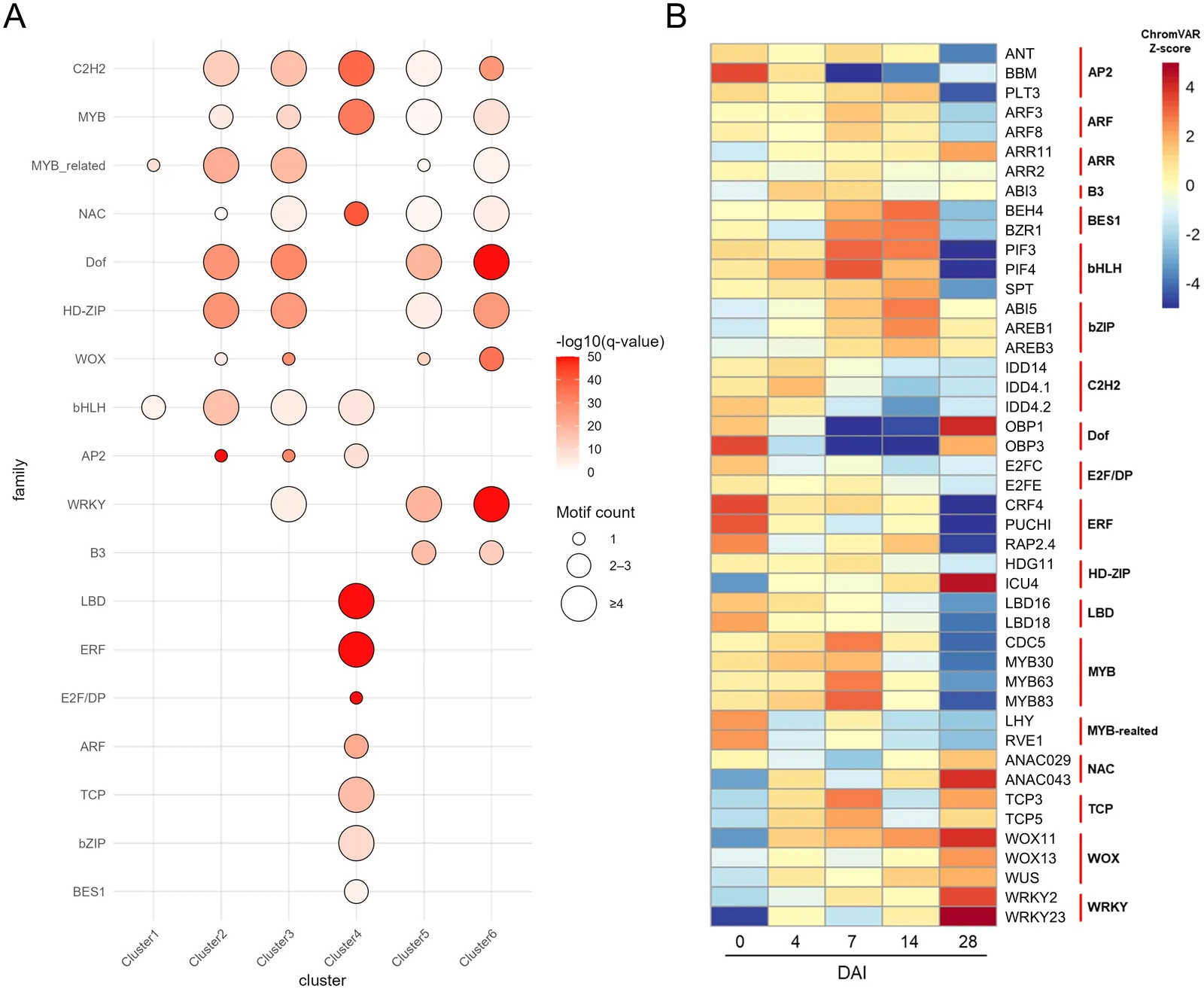
Temporal dynamics of transcription factor binding and motif activity during callus development. **(A)** Cluster-based motif enrichment analysis of open chromatin regions (OCRs). Dot plots display significantly enriched transcription factor (TF) families across the six DAR clusters. Dot size indicates the number of enriched motifs within each TF family, and the color gradient represents enrichment significance [-log_10_(*q*-value)]. **(B)** Temporal dynamics of TF binding motif activity estimated using chromVAR. Heatmaps show Z-score-standardized chromVAR deviation scores for representative TF motifs across developmental stages (days after induction [DAI] 0, 4, 7, 14, and 28). ChromVAR deviation scores represent relative chromatin accessibility at TF-binding motifs. Values were Z-score standardized for visualization.

In addition, enrichment of ERF (Ethylene Response Factor) family motifs, including *WIND1*, suggests a potential contribution of wound-induced ethylene signaling to early dedifferentiation and cell wall remodeling. E2F/DP motifs, which are associated with DNA replication and G1/S cell cycle progression, together with BES1/BZR motifs involved in brassinosteroid-mediated growth and cell wall synthesis, were enriched during the callus-log stage, consistent with coordinated involvement of proliferative and growth-related pathways. Enrichment of TCP and bZIP motifs further implies involvement of higher-order regulatory mechanisms associated with chromatin organization and transcriptional coordination (Karaaslan et al., 2020).

In contrast, WRKY motifs were predominantly enriched in Clusters 3, 5, and 6 during the stationary stage (DAI 14–28). Because WRKY TFs are well known regulators of ABA-, ROS-, and defense-associated signaling pathways (Wani et al., 2021), these patterns suggest increased involvement of stress-adaptive transcriptional programs during late callus development.

Temporal TF motif-associated chromatin accessibility was further evaluated using chromVAR (**Figure 5B**). During the callus-log stage (DAI 4–7), ARF, LBD, ERF, and E2F/DP motifs exhibited high deviation scores that progressively declined during the transition to the stationary stage. ARF and LBD motifs, key regulators of callus induction, exhibited progressively decreasing deviation scores after DAI 7 during the transition to the stationary stage, suggesting that auxin-associated regulatory activity became less prominent during the stationary stage. Similarly, BES1 and ERF motifs exhibited a marked decline by DAI 28, suggesting reduced association of brassinosteroid- and wound-responsive regulatory pathways during late callus development. In contrast, WOX motifs maintained relatively high deviation scores after DAI 14, consistent with a potential role in maintaining the undifferentiated state of callus tissues. Similarly, WRKY motifs exhibited increased deviation scores during the later stages, consistent with increased association with stress- and defense-related regulatory programs during the stationary stage.

Together, these findings indicate temporal shifts in TF motif enrichment and motif-associated chromatin accessibility during soybean callus development, with distinct TF groups preferentially associated with the early callus-log stage and the later stationary stage. These temporal patterns coincided with stage-specific changes in chromatin accessibility, suggesting distinct stage-dependent TF-associated regulatory signatures.

### 3.6 Gene regulatory network analysis and temporal coordination between transcription factors and developmental programs

To further define the regulatory architecture underlying soybean callus development, we constructed OCR-based gene regulatory networks (GRNs) by integrating chromatin accessibility with TF binding information. Based on previously characterized developmental pathways and motif annotations from the PlantTFDB, nine representative TFs were selected for GRN analysis: *PLT3*, *WOX11*, *LBD16*, *LBD18*, *E2FC*, *E2FE*, *OBP1*, *NAC071*, and *WIND1*.

Temporal expression profiling of these TFs revealed sequential and continuous expression dynamics across the pre-callus, callus-log, and callus-stationary stages. In the rlog-transformed data (**Figure 6A**), *PLT3* maintained consistently high absolute expression throughout development, suggesting sustained functional importance during early callus development. Similarly, *WIND1*, which exhibited elevated expression during the stationary stage, also maintained high transcript abundance throughout development. In the Z-score-scaled analyses (**Figure 6B**), however, *PLT3* showed reduced relative expression after induction.

**Figure 6:**
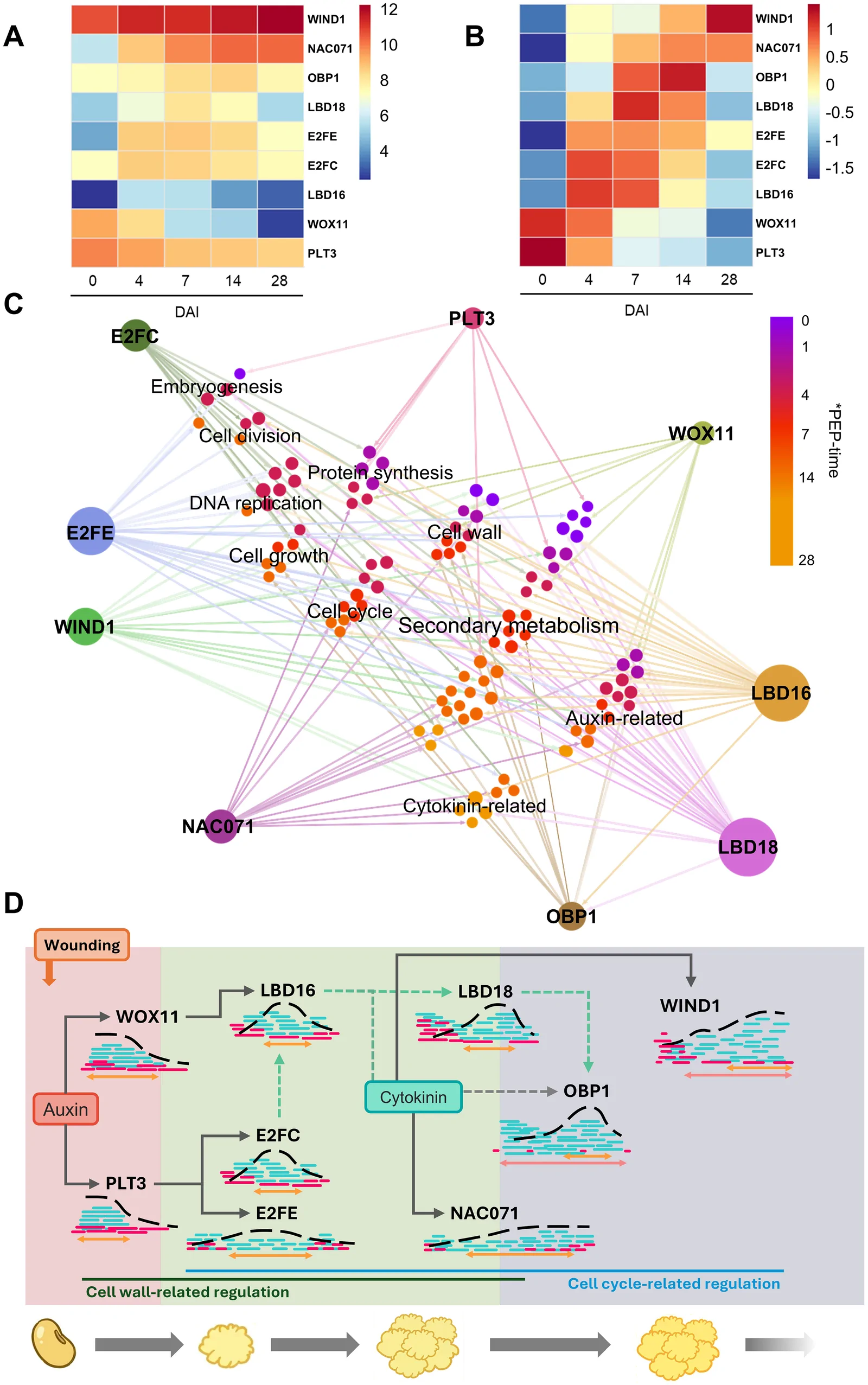
Gene regulatory network analysis of representative transcription factors during soybean callus development. **(A)** non-scaled regularized log (rlog) expression profiles and **(B)** Z-score-scaled expression profiles of representative TFs during callus development. **(C)** Gene regulatory network (GRN) constructed from open chromatin region (OCR)-associated TF-binding prediction. PEP-time indicates pseudo-expression peak time, and target genes are grouped according to enriched GO biological processes. **(D)** Schematic model summarizing transcriptional and epigenomic dynamics associated with nine representative transcription factors during callus induction and proliferation. Grey arrows indicate experimentally supported regulatory relationships, whereas dashed green arrows indicate putative regulatory interactions inferred from network analysis. PEP-time indicates the day after induction (DAI) at which the average expression of each target gene group reaches its maximum. Pale red, green, and blue backgrounds represent the pre-callus, callus-log and callus-stationary stages, respectively. Cyan and magenta bars indicate activating and repressive epigenomic signals, respectively. LBD, lateral organ boundaries domain; WOX, WUSCHEL-like homeobox; PLT, PLETHORA; E2F, E2F transcription factor; *NAC071*, NAC domain-containing protein 71; *OBP1*, OBF-binding protein 1; *WIND1*, WOUND INDUCED DEDIFFERENTIATION 1.

GRN construction focused on these nine TFs. FIMO-based motif scanning within union OCRs identified 128,840 candidate TF-binding sites, which were narrowed to 25,005 high-confidence sites after significance filtering (FIMO p < 1×10^−5^). Assigning these sites to gene promoters has retained 18,088 promoter-associated binding sites for network construction. The resulting candidate TF–target associations were subjected to GO enrichment analysis. Callus-associated GO categories were subsequently integrated to generate functional regulatory subnetworks grouped according to biological processes and temporal expression patterns (**Figure 6C**; **Supplementary Table 6**).

Distinct developmental programs displayed coordinated temporal activation patterns. Gene sets associated with cell wall organization, cell growth, and cell division were activated predominantly during the callus-log stage, whereas genes involved in cell cycle progression and sustained growth remained highly expressed into the stationary stage. Among hormone-responsive pathways, auxin-associated genes were activated earlier than cytokinin-responsive genes, indicating sequential hormonal regulation during callus development. Genes related to secondary metabolism exhibited broad activity across both early and late developmental stages.

To further examine temporal regulatory coordination, TF-associated GO groups were arranged according to pseudo-expression peak time (PEP-time; **Figure 6B**). This analysis revealed strong concordance between TF expression timing and the activation patterns of their predicted target genes. Early-stage TFs such as *PLT3* and *WOX11* were associated with GO groups maximally expressed at DAI 0 and 4. Predicted *PLT3* target genes were enriched for functions related to protein biosynthesis, consistent with rapid proliferative activity, whereas predicted *WOX11* target genes were associated with auxin responses and cell wall remodeling during early dedifferentiation.

In contrast, *LBD16*, *LBD18*, and *E2FC* were linked to target gene sets peaking during DAI 1–7, whereas *E2FE*, *OBP1*, and *NAC071* were associated with later-expressed gene groups peaking at DAI 4–14. Notably, *NAC071* was preferentially associated with late-stage genes related to cytokinin signaling and cell cycle regulation, suggesting a role in sustaining proliferative activity during callus maturation.

Interestingly, *WIND1* exhibited elevated expression during the stationary stage (**Figure 6A**), while its predicted target set included both early-response genes involved in auxin signaling, protein synthesis, and cell wall organization, and late-stage genes associated with cytokinin signaling and cell cycle regulation. Among the nine TFs, *WIND1* showed the broadest and most even distribution of predicted targets across the eight temporal expression clusters (highest target-cluster Shannon entropy, 2.96 of a maximum 3.0; **Supplementary Figure 8**). This temporal breadth, rather than the highest overall connectivity, distinguishes *WIND1* as a candidate coordinator spanning early- and late-stage callus programs. Together with its own progressively increasing expression, these observations suggest that *WIND1* may be particularly associated with the maintenance of multiple developmental programs during the later stages of callus development.

Overall, these results indicate temporal associations between TF expression patterns and chromatin accessibility dynamics at predicted target-associated OCRs. Based on these relationships, we constructed an integrated regulatory model linking epigenomic remodeling, TF activity, and transcriptional reprogramming during soybean callus development (**Figure 6D**; **Supplementary Figure 9**).

## 4 Discussion

### 4.1 Hierarchical epigenetic reprogramming during callus formation

Our previous transcriptomic study demonstrated that soybean callus formation involves extensive temporal reprogramming associated with hormonal signaling, cell cycle activation, and developmental transitions (Park et al., 2023). However, the chromatin-level mechanisms coordinating these transcriptional changes remained unresolved. In the present study, integration of ATAC-seq and ChIP-seq datasets with transcriptomic profiles provides evidence that soybean callus development is accompanied by hierarchical epigenetic reprogramming involving coordinated changes in chromatin accessibility and histone modification. Indeed, principal component analysis across all multi-omics datasets showed that the largest separation occurred between DAI 0 and DAI 4, suggesting that the transition from DAI 0 to DAI 4 is associated with the most extensive epigenomic and transcriptional reorganization during callus induction. The additional separation observed between DAI 4 and DAI 7 specifically in the ATAC-seq dataset further suggests that chromatin accessibility continues to undergo dynamic remodeling following the initial reprogramming phase.

These observations are consistent with the stage-dependent chromatin remodeling observed during callus development. The transition from differentiated explant tissue to a pluripotency-competent callus state was characterized by sequential chromatin remodeling events. Early callus induction involved repression of explant-associated chromatin states, as reflected by reduced accessibility in regions linked to photosynthesis and tissue-specific developmental programs. This was followed by extensive chromatin opening at the regulatory regions associated with hormone signaling, developmental regulators, and proliferative pathways. Subsequently, chromatin states became progressively stabilized during the stationary stage (**Figure 2**). These stage-dependent changes indicate that callus formation is not driven by a single transcriptional switch, but rather by a coordinated progression of epigenetic transitions accompanying cellular dedifferentiation and establishment of a new developmental identity (Liu et al., 2023b; Li et al., 2024).

Importantly, our analyses revealed that dynamic increases in chromatin accessibility were closely associated with H3K27ac accumulation and transcriptional activation during the early induction stage (**Figures 3**, **4**). In contrast, H3K27me3-associated regions exhibited higher chromatin-state plasticity and frequent transitions during early reprogramming (Du et al., 2013; Oka et al., 2017). Together, our findings suggest that chromatin remodeling is closely associated with transcriptional reprogramming during soybean callus formation and may provide an epigenomic framework for these transcriptional transitions.

### 4.2 Dynamic chromatin accessibility defines distinct stages of cell-fate transition

Clustering analysis of differentially accessible regions (DARs) revealed that chromatin accessibility dynamics closely correspond to distinct developmental and physiological stages during callus formation (**Figure 2**). These accessibility patterns provide insight into how cellular identity is progressively reconfigured during dedifferentiation.

During the early induction stage (DAI 0–4), accessibility rapidly decreased in Clusters 1 and 2, which were enriched for genes associated with photosynthesis and organ development. This broad reduction in accessibility suggests that repression of pre-existing somatic identity is an essential prerequisite for cellular dedifferentiation. Such observations are consistent with the concept that acquisition of pluripotency requires attenuation of differentiated cell programs before activation of regenerative pathways (Fehér, 2019).

In contrast, the callus-log stage (DAI 4–7)was characterized by pronounced chromatin opening in Cluster 4. These accessible regions were strongly associated with auxin- and cytokinin-responsive genes, including members of the *ARF* and *ARR* families, together with developmental regulators such as *LBD* genes and *WIND1*. Importantly, these chromatin changes coincided with activation of genes involved in cell wall remodeling and cell-cycle progression, suggesting an association among hormonal and wound-responsive signaling, chromatin remodeling, and proliferative reprogramming during early callus establishment (Ikeuchi et al., 2013; Kareem et al., 2015).

During stationary stages (DAI 14–28), accessibility increased predominantly in Clusters 3 and 6, which were enriched for ABA signaling, ROS regulation, and stress- and defense-related pathways. These findings suggest that the stationary stage involves a shift from active proliferative reprogramming toward maintenance and environmental adaptation. Because long-term callus culture is often associated with nutrient limitation and oxidative stress, activation of stress-responsive chromatin programs may contribute to sustaining cellular viability under prolonged culture conditions (Wani et al., 2021; Wan et al., 2025). Meanwhile, in contrast to the delayed chromatin opening observed in Cluster 6, the sustained accessibility of Cluster 5 from DAI 4 through the stationary stage suggests an association with the maintenance of callus identity and continued developmental competence.

Together, these findings indicate that dynamic chromatin accessibility is closely associated with the progression from somatic identity suppression to proliferative dedifferentiation and ultimately to stabilized callus maintenance.

### 4.3 Temporal decoupling between histone modifications and transcription during callus development

Integration of chromatin accessibility, histone modification, and transcriptomic datasets revealed that the relationship between epigenetic marks and gene expression profiles changes substantially across developmental stages. During the early induction phase (DAI 0–7), H3K27ac accumulation showed strong positive associations with both chromatin accessibility and transcriptional activation, whereas H3K27me3 was negatively correlated with gene expression (**Figures 3A, B**). These relationships are consistent with the established roles of H3K27ac and H3K27me3 as activating and repressive chromatin marks, respectively (Li et al., 2024).

Notably, however, these correlations became substantially weaker during the later stages of callus development (DAI 7–28). Although chromatin accessibility remained strongly associated with transcriptional output throughout development, the direct relationship between histone modifications and gene expression progressively diminished. This temporal decoupling suggests that histone modifications may play a more prominent role during the initiation of transcriptional reprogramming, whereas maintenance of transcriptional states may depend on additional regulatory mechanisms.

One possible explanation is that once accessible chromatin states and transcriptional networks are established during early callus induction, gene expression becomes increasingly stabilized through multilayered regulatory systems involving additional histone modifications, DNA methylation, chromatin topology, and transcription factor network interactions (Klemm et al., 2019). Consistent with this interpretation, H3K27ac exhibited strong coupling with OCR accessibility specifically during the early phase(**Figures 3C, D**), suggesting a close association between enhancer/promoter-associated chromatin changes and the onset of dedifferentiation. In contrast, later developmental stages appear to reflect maintenance of pre-established regulatory states rather than ongoing large-scale chromatin activation.

These findings highlight the dynamic and stage-dependent epigenetic reprogramming during callus formation and suggest that distinct chromatin features contribute differently across developmental stages.

### 4.4 Sequential interactions in transcription factor networks coordinate regulatory transitions during callus formation

Integration of motif enrichment (MEME-AME) and chromVAR analyses provided complementary information regarding temporal changes in TF motif representation and motif-associated accessibility during soybean callus development (**Figure 5**). The results are consistent with a sequential model in which distinct TF-associated motif activities emerge at specific developmental stages and are associated with the transition from differentiated explant tissue to pluripotent callus.

During the early induction stage (DAI 0–4), enrichment of ERF and ARF–LBD motifs within newly accessible chromatin regions suggest that wound-responsive and auxin-mediated signaling pathways are closely associated with early cellular reprogramming during callus induction (Iwase et al., 2011; Liu et al., 2016). In particular, enrichment of *LBD16* and *LBD18* motifs, together with increased accessibility of auxin-responsive loci, supports a close association between auxin-responsive chromatin remodeling and dedifferentiation-associated transcriptional programs. Similarly, enrichment of ERF-family motifs, including WIND1, is consistent with a potential contribution of wound signaling to early chromatin reorganization and developmental transition (**Figure 5**).

During the callus-log stage (DAI 4–7), elevated activity of E2F/DP and BES1 motifs was associated with genes involved in cell cycle progression, and cell wall biogenesis. These findings suggest coordinated involvement of proliferative growth through integration of cell-cycle regulators and brassinosteroid signaling pathways (Inzé and De Veylder, 2006; Wolf et al., 2012; Sun et al., 2010). Elevated chromatin accessibility and motif activity during this stage likely reflect a highly permissive regulatory environment supporting rapid cell proliferation and expansion.

At later stages (DAI 14–28), sustained activity of WOX motifs was associated with maintenance of the undifferentiated callus state, whereas increased activity of WRKY motifs correlated with stress- and defense-related transcriptional programs. These observations indicate a progressive shift from active developmental reprogramming toward stabilization and environmental adaptation of callus tissues (Hu and Xu, 2016; Kong et al., 2016; Khoso et al., 2022; Huang et al., 2024; Boonyaves et al., 2022).

Importantly, OCR-based gene regulatory network analysis further identified predicted TF-associated regulatory modules linked to different stages of soybean callus development. Sequential changes in predicted TF-associated modules, including *PLT3*, *WOX11*, *LBD16/18*, *E2FC/E2FE*, *NAC071*, and *WIND1*, were linked to distinct developmental processes such as protein biosynthesis, auxin signaling, cell wall remodeling, cell-cycle progression, and cytokinin responses (**Figure 6B**). Among these regulators, *WIND1* was notable not for the largest number of target connections but for the breadth of its predicted targets, which were the most evenly distributed across early- to late-stage expression programs among the nine TFs (**Supplementary Figure 8**). Together with its progressively increasing expression toward the stationary stage, this pattern suggests that *WIND1* may represent a candidate temporal coordinator linking early wound-responsive pathways with later proliferative and maintenance-associated programs.

Despite these insights, the gene regulatory networks reconstructed in this study remain computational predictions rather than experimentally validated regulatory relationships. They were inferred from TF motif occurrence within promoter-associated OCRs, peak-to-gene proximity, and expression-based filtering; consequently, they may contain false-positive TF–target associations and do not provide direct evidence of physical TF binding or of the causal direction of regulation. Motif-based prediction cannot distinguish functional binding events from incidental motif matches, and shared chromatin accessibility or co-expression does not by itself establish direct regulation. Accordingly, specific inferences drawn from these networks, including the proposed role of *WIND1* as a temporal coordinator of early- and late-stage callus programs, remain bioinformatic predictions rather than established regulatory functions. Experimental approaches such as DAP-seq, TF ChIP-seq, or CRISPR-based perturbation will be required to validate these predicted interactions. The networks presented here should therefore be regarded as a hypothesis-generating framework that prioritizes candidate regulators and target programs for future functional investigation.

Overall, this study provides a genome-wide framework describing the epigenetic and transcriptional regulatory dynamics underlying soybean callus formation. Coordinated remodeling of chromatin accessibility and histone modifications, together with temporally associated changes in TF motif-associated activity, is consistent with a stepwise transition from differentiated somatic identity to a pluripotent callus state. These findings provide valuable insight into the molecular basis of plant cellular reprogramming and establish candidate regulatory elements and transcription factors that may be targeted to improve regeneration efficiency and tissue culture performance in soybean.

## 5 Conclusion

This study provides a genome-wide epigenomic framework for soybean callus development through integration of chromatin accessibility, histone modification, and transcriptomic analysis. Our results demonstrate that callus formation is accompanied by hierarchical epigenetic reprogramming, in which dynamic changes in chromatin accessibility are closely associated with transcriptional activation during early cellular dedifferentiation and proliferative reprogramming. In particular, the formation of *de novo* open chromatin regions was associated with increased accessibility and expression of key developmental regulators and hormone-responsive genes, while temporal changes in TF motif enrichment and motif-associated chromatin accessibility identified candidate stage-associated regulatory signatures during callus development. Collectively, these findings provide new insights into chromatin-mediated regulation of plant cellular reprogramming and establish a foundation for improving regeneration efficiency and tissue culture-based crop biotechnology in soybean.

## Data Availability

The data presented in the study are deposited in the NCBI BioProject repository, accession numbers PRJNA949161 (RNA-seq) and PRJNA1457011 (ATAC-seq and ChIP-seq): https://www.ncbi.nlm.nih.gov/bioproject/PRJNA949161 and https://www.ncbi.nlm.nih.gov/bioproject/PRJNA1457011.
