# Supplementary material for: Coordinated epigenomic reprogramming and chromatin-mediated transcriptional regulation during soybean callus development
Source: Front Plant Sci. 2026 Sep 25;17:1905128. doi: 10.3389/fpls.2026.1905128 (PMC13649698; doi:10.3389/fpls.2026.1905128)
Supplement: Supplementary file 1 [file DataSheet1.zip › Supplementary Figure.PDF]

# *Supplementary Material*

## 1 Supplementary Figures

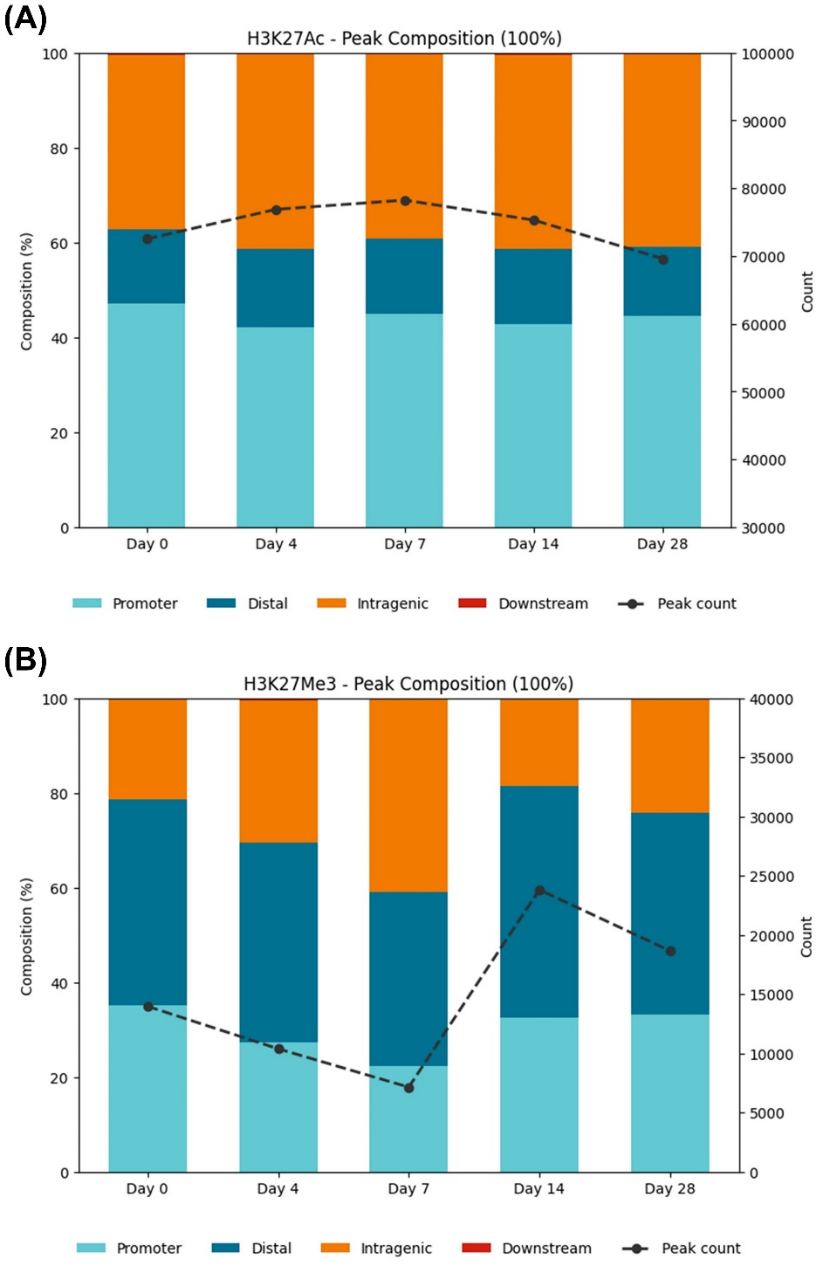


**Supplementary Figure 1. Genome-wide distribution of histone modification marks.** Each plot represents (A) H3K27ac and (B) H3K27me3 at each time point. Stacked bars show genomic composition (%), including promoter, distal, intragenic, and downstream regions, and the dashed line represents peak counts.

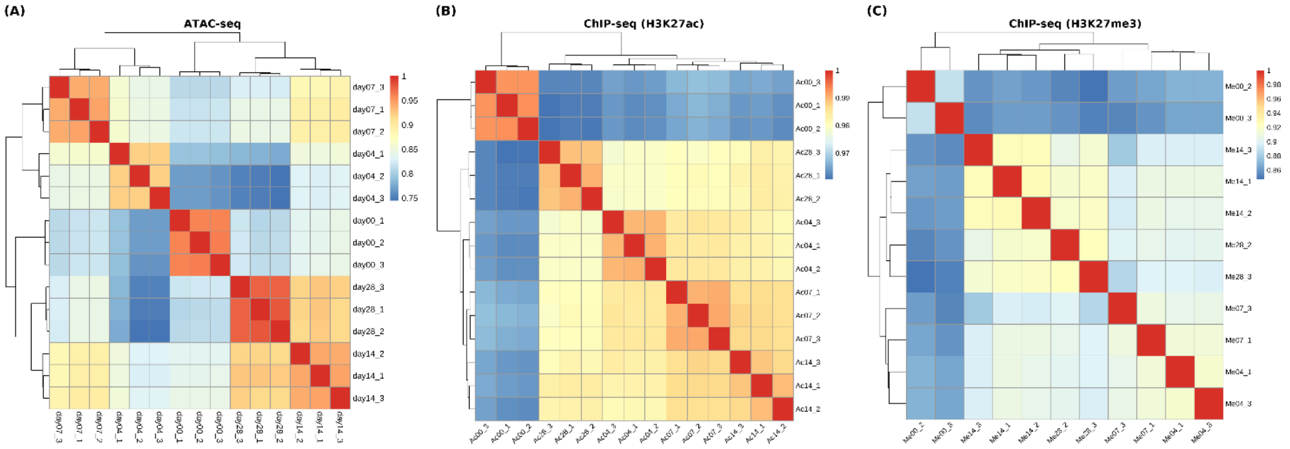


**Supplementary Figure 2. Correlation heatmaps of ATAC-seq and ChIP-seq samples.** Correlation heatmaps for ATAC-seq (a) and ChIP-seq (H3K27ac, H3K27me3) (b, c). Each sample includes 2–3 biological replicates.

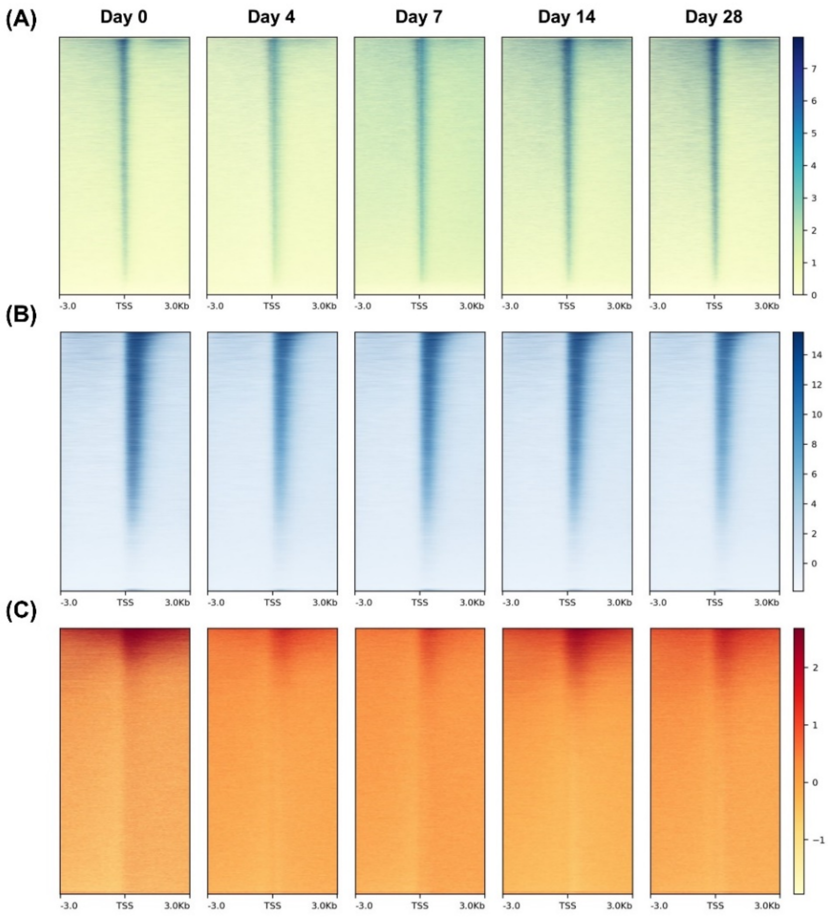


**Supplementary Figure 3. Signal distribution of ATAC-seq and ChIP-seq.** Genome-wide signal profiles centered on transcription start sites (TSSs; ±3 kb) across five time points (DAI 0, 4, 7, 14, and 28). (A) ATAC-seq signal showing enrichment of chromatin accessibility around TSS regions. (B) ChIP-seq signal for H3K27ac displaying enrichment at promoter and genic regions. (C) ChIP-seq signal for H3K27me3 showing broader distribution across gene bodies.

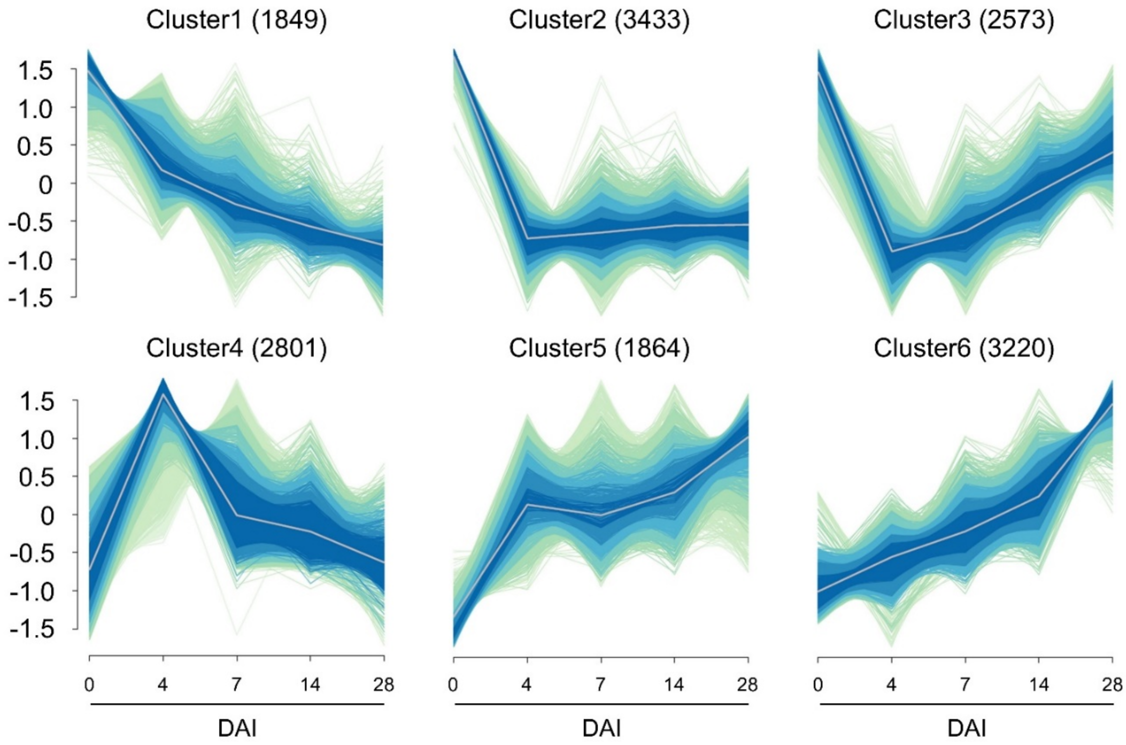


**Supplementary Figure 4. Visualization of OCR accessibility cluster as line plot.**

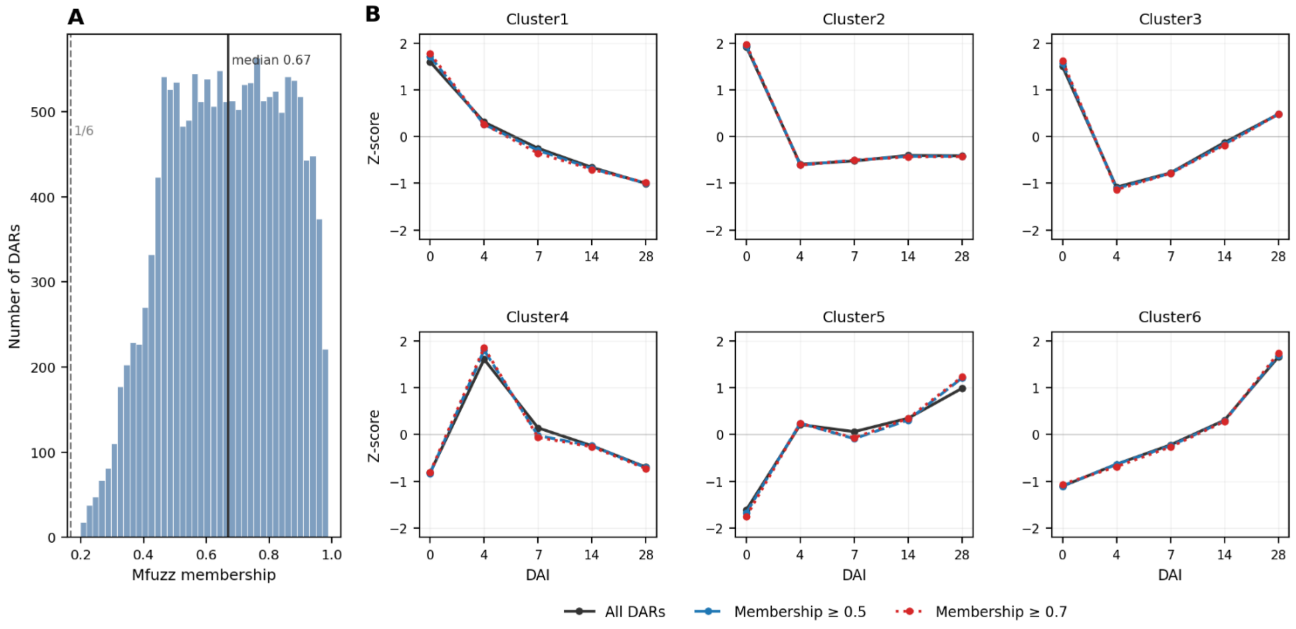


**Supplementary Figure 5. Mfuzz membership values and cluster profiles at increasing membership cut-offs.** (A) Distribution of membership values for the 15,740 differentially accessible regions (DARs); solid line, median; dashed line, the value expected at random for six clusters. (B) Mean Z-score profile of each cluster across the five time points, computed from all DARs and from members with membership ≥ 0.5 or ≥ 0.7. DAI, days after induction.

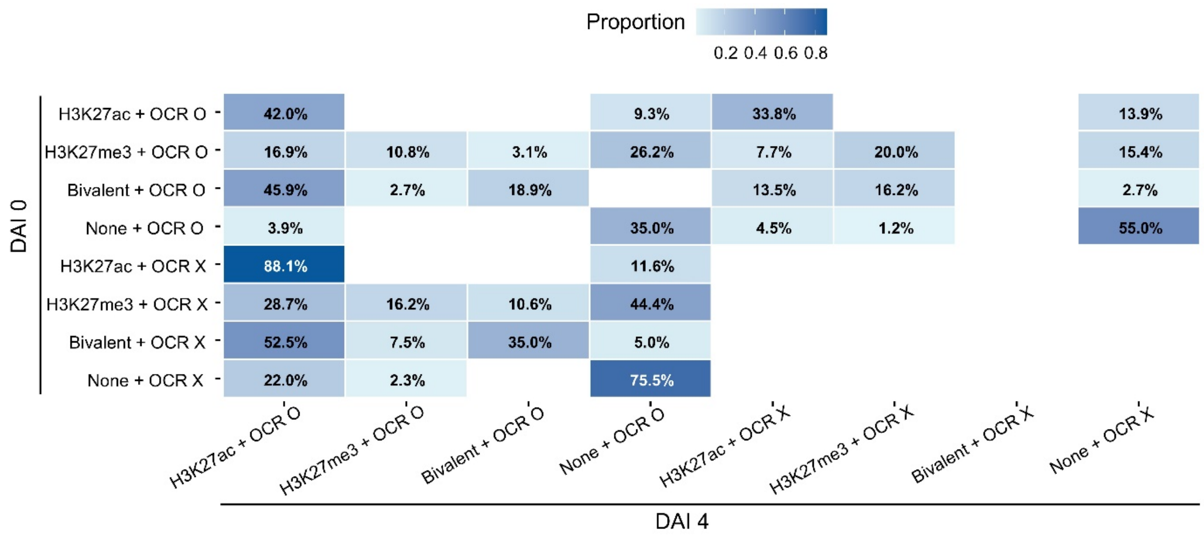


**Supplementary Figure 6. Histone mark profile during transition from explant to callus.**

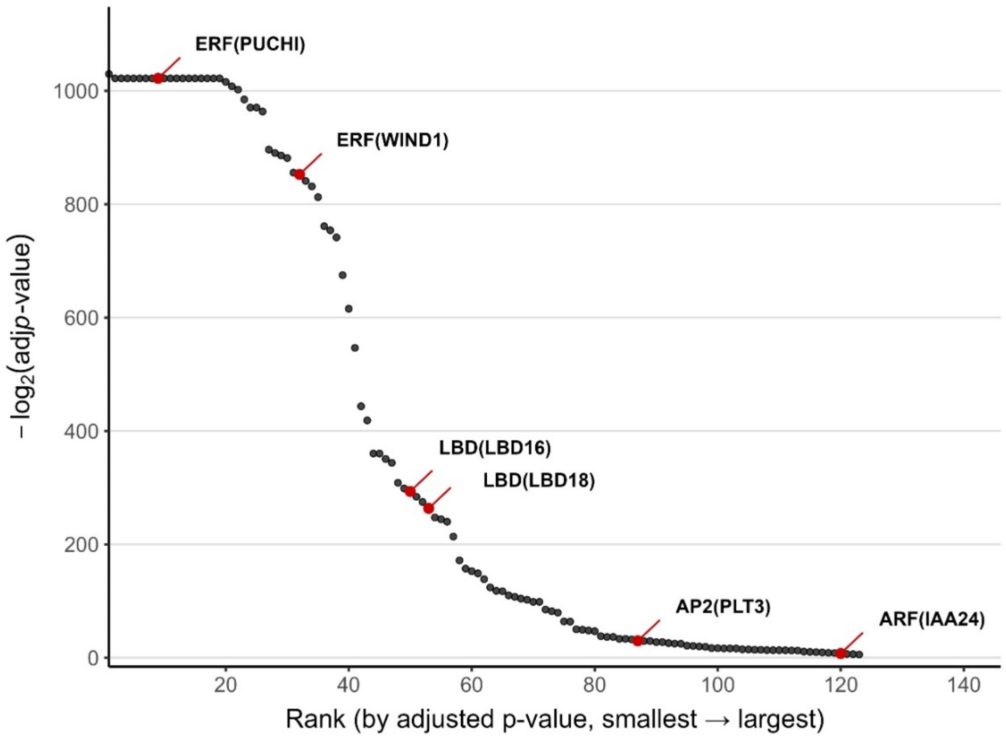


**Supplementary Figure 7. Identification of highly enriched TF-binding motifs within open chromatin regions in cluster 4.**

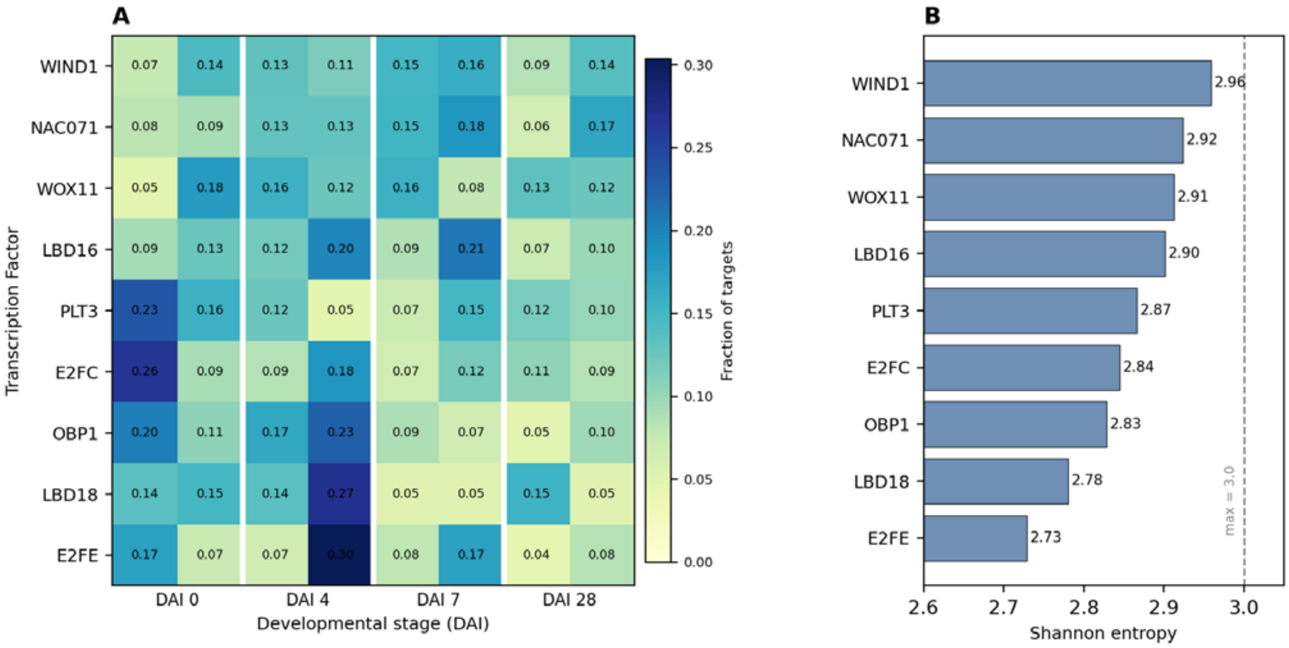


**Supplementary Figure 8. Temporal distribution of predicted target genes for the 9 transcription factors.** (A) Fraction of each TF's target genes assigned to the eight expression clusters (Park et al., 2023), grouped by peak stage (DAI; two clusters per stage). (B) Shannon entropy of each TF's target distribution across the eight clusters (dashed line, maximum = $\log_2 8 = 3.0$).

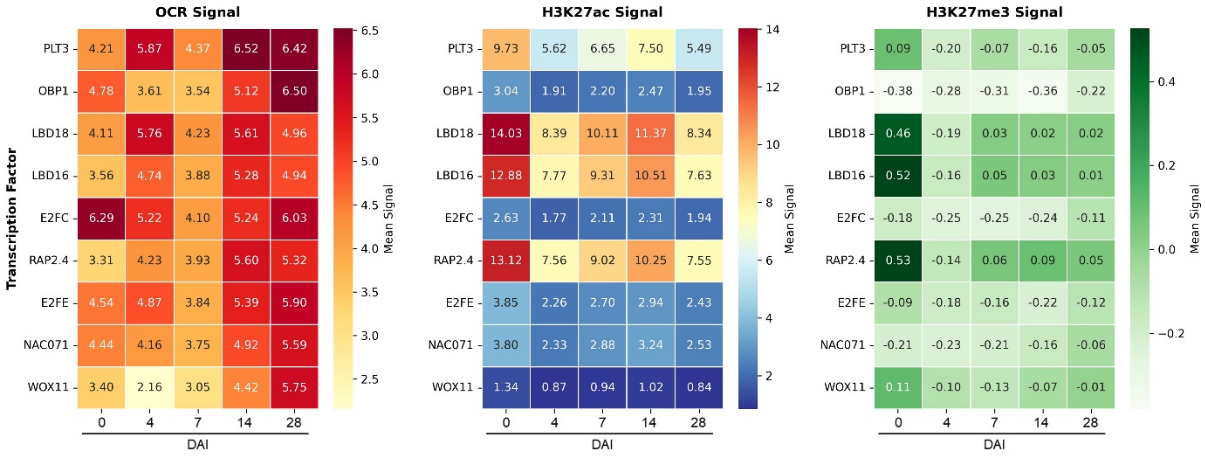


**Supplementary Figure 9. Average epigenomic signal amount binding sites of 9 transcription factors.**
